# Smad7 deficiency decreases iron and haemoglobin through hepcidin up‐regulation by multilayer compensatory mechanisms

**DOI:** 10.1111/jcmm.13546

**Published:** 2018-03-25

**Authors:** Peng An, Hao Wang, Qian Wu, Jiaming Wang, Zhidan Xia, Xuyan He, Xinhui Wang, Yan Chen, Junxia Min, Fudi Wang

**Affiliations:** ^1^ Beijing Advanced Innovation Center for Food Nutrition and Human Health China Agricultural University Beijing China; ^2^ School of Public Health, The First Affiliated Hospital Institute of Translational Medicine Collaborative Innovation Center for Diagnosis and Treatment of Infectious Diseases School of Medicine Zhejiang University Hangzhou China; ^3^ Precision Nutrition Innovation Center School of Public Health Zhengzhou University Zhengzhou China; ^4^ Key Laboratory of Nutrition and Metabolism Institute for Nutritional Sciences Shanghai Institutes for Biological Sciences Chinese Academy of Sciences University of Chinese Academy of Sciences Shanghai China

**Keywords:** *Bambi*, follistatin, hepcidin, iron deficiency, *Smad6*, *Smad7*

## Abstract

To maintain iron homoeostasis, the iron regulatory hormone hepcidin is tightly controlled by BMP‐Smad signalling pathway, but the physiological role of Smad7 in hepcidin regulation remains elusive. We generated and characterized hepatocyte‐specific *Smad7* knockout mice (*Smad7* ^*Alb/Alb*^), which showed decreased serum iron, tissue iron, haemoglobin concentration, up‐regulated hepcidin and increased phosphor‐Smad1/5/8 levels in both isolated primary hepatocytes and liver tissues. Increased levels of hepcidin lead to reduced expression of intestinal ferroportin and mild iron deficiency anaemia. Interestingly, we found no difference in hepcidin expression or phosphor‐Smad1/5/8 levels between iron‐challenged *Smad7* ^*Alb/Alb*^ and *Smad7* ^*flox/flox*^, suggesting other factors assume the role of iron‐induced hepcidin regulation in Smad7 deletion. We performed RNA‐seq to identify differentially expressed genes in the liver. Significantly up‐regulated genes were then mapped to pathways, revealing TGF‐β signalling as one of the most relevant pathways, including the up‐regulated genes *Smad6*,* Bambi* and *Fst* (Follistatin). We found that Smad6 and Bambi—but not Follistatin—are controlled by the iron‐BMP–Smad pathway. Overexpressing Smad6, Bambi or Follistatin in cells significantly reduced hepcidin expression. Smad7 functions as a key regulator of iron homoeostasis by negatively controlling hepcidin expression, and Smad6 and Smad7 have non‐redundant roles. Smad6, Bambi and Follistatin serve as additional inhibitors of hepcidin in the liver.

## 1 INTRODUCTION

Maintaining iron homoeostasis is essential for maintaining normal cellular function. To avoid pathological iron overload and/or deficiency, iron levels are tightly regulated by the liver‐derived peptide hepcidin.^1^, ^2^ At the systemic level, hepcidin maintains iron homoeostasis by binding to and degrading the protein ferroportin, the sole exporter of cellular iron.^3^ Conversely, both circulating iron and tissue iron provide specific signals that modulate hepcidin expression.

Perturbations in hepcidin expression can lead to a variety of iron‐related disorders. For example, reduced hepcidin level causes iron overload in hereditary haemochromatosis (HH) and iron‐loading anaemia, which is induced by ineffective erythropoiesis.^4^ In HH types I, II and III, mutations either in the hepcidin‐encoding gene *HAMP* or in genes that encode hepcidin regulators can reduce the expression of hepcidin, thereby increasing duodenal iron absorption and causing clinical iron overload.^5^, ^6^, ^7^ In contrast, increased hepcidin expression causes iron restriction in a variety of inflammatory conditions, including autoimmune disease, critical illness, certain types of cancers and chronic kidney disease.^8^ Therefore, considerable effort has been devoted to developing agents that target hepcidin and/or its regulators in order to develop novel therapeutic strategies for treating iron‐related disorders.^9^ In addition, hepcidin and hepcidin agonists can exert a protective effect on the liver, heart and other vital organs by redistributing iron into macrophages in the liver and spleen. Thus, given the high therapeutic potential of hepcidin, understanding how hepcidin is regulated in vivo is essential.

In hepatocytes, hepcidin expression is regulated by the BMP‐Smad signalling pathway. Binding of BMP ligands (eg BMP6) to BMP receptors on the surface of hepatocytes triggers the downstream phosphorylation of Smad proteins.^10^, ^11^ Under dietary iron stimulation, hepatic BMP6 triggers the phosphorylation of Smad1/5/8, together with Smad4, to translocate to the nucleus, where they activates hepcidin expression.^12^ Therefore, both *Bmp6*‐deficient mice and mice with liver‐specific *Smad4* deletion have reduced hepcidin expression and develop an severe iron‐overload phenotype.^12^, ^13^ Results obtained from studying patients with HH types I, II or III—together with their corresponding genetic mouse models—support the notion that defective BMP‐Smad signalling leads to hepcidin insufficiency.^6^, ^7^, ^14^, ^15^, ^16^, ^17^

Smad7 is a negative regulators of BMP‐Smad signalling, and the function of Smad7 protein in iron metabolism is poorly understood, although a growing body of in vitro evidence supports the notion that inhibitory Smads regulate hepcidin expression.^18^, ^19^ Based on a genomewide liver transcription profiling study, the expression of *Smad7* was found to be up‐regulated by iron‐enriched diet.^20^ However, whether—and how—the Smad7 regulates dietary iron intake and hepcidin expression in the liver is currently unknown. Therefore, in this study, we generated and characterized a hepatocyte‐specific *Smad7*‐knockout mouse model to investigate the physiological role of Smad7 in regulating iron metabolism.

## 2 MATERIALS AND METHODS

### 2.1 Animals and treatments

Conditional *Smad7‐floxed* 21 mice were backcrossed with wild‐type C57BL/6 mice (SLRC Laboratory Animal Co., Ltd., Shanghai, China) for at least seven generations, then crossed with albumin‐Cre (Alb) transgenic mice (on a C57BL/6 background) to obtain hepatocyte‐specific *Smad7*‐knockout (*Smad7* ^*Alb/Alb*^) mice. The *Smad7*‐knockout mice used in this study were 8 week old of littermates. *Hfe* ^*−/−*^ mice were kindly provided by Dr. Nancy C. Andrews,^22^ and *Smad4* ^*Alb/Alb*^ mice were kindly provided by Dr. Chu‐xia Deng.^12^ The *Hfe* ^*−/−*^ and *Smad4* ^*Alb/Alb*^ mice were maintained on the 129/SvEvTac background, and 8‐week‐old mice were used in this study. All mice were housed under specific pathogen‐free conditions and fed a standard rodent diet (SLRC Laboratory Animal Co., Ltd, Shanghai, China) containing 232 mg/kg iron.^23^ The iron‐rich diet used for the iron‐challenged experiments was composed of standard diet containing 8.3 g/kg carbonyl iron. All animal protocols were approved by the Animal Studies Committee of Zhejiang University and the Institute for Nutritional Sciences, Shanghai Institutes for Biological Sciences, Chinese Academy of Sciences.

### 2.2 Measurement of haematological parameters, serum iron and tissue non‐haem iron

Whole blood (100 μL) was obtained by cardiac puncture and collected in a tube containing the anticoagulant ethylenediaminetetraacetic acid (EDTA). Haematological parameters were measured at the Xuhui District Central Hospital (Shanghai, China) using a Sysmex XS‐800i Automated Hematology Analyzer (Sysmex Corporation, Kobe, Japan). Serum iron (SI) and unsaturated iron‐binding capacity (UIBC) were measured using a commercially available colorimetry‐based detection kit (Pointe Scientific). Total iron‐binding capacity (TIBC) and transferrin saturation (TS) were calculated from SI and UIBC as follows: TIBC = SI + UIBC and TS = (SI/TIBC × 100). Tissue non‐haem iron concentration was measured as previously described.^24^

### 2.3 Ferroportin immunohistochemistry

Intestinal ferroportin detection using immunohistochemistry and Perls’ Prussian blue iron staining was performed as previously described.^23^

### 2.4 Isolation and culture of primary hepatocyte

Primary hepatocytes were isolated as previously described,^25^ cultured for 16 hours in Dulbecco's modified Eagle's medium (DMEM, Gibco) containing 10% foetal bovine serum (v/v), and then collected for experiments. Where indicated, the cells were cultured with human recombinant BMP6 (R&D Systems) and/or human holo‐Transferrin (Sigma‐Aldrich).

### 2.5 Plasmid generation and overexpression in cell lines

The open reading frames of the *Smad6*,* Smad7*,* Bambi* and *Fst* mRNAs (NCBI reference sequences NM_005585.4, NM_001042660.1, NM_012342.2 and NM_006350.3, respectively) were amplified from a cDNA library of the HepG2 cell line and inserted into the pCMV‐3tag‐3A vector (Stratagene). All constructs and their protein products were confirmed using DNA sequencing and Western blot analysis, respectively. Huh7 cells, a human hepatoma cell line, were plated in 12‐well plates and cultured at 37°C in 5% CO_2_ with 1 mL/well DMEM (Gibco) containing 15% (v/v) heat‐inactivated foetal bovine serum (Gibco). The cells were then transfected with the respective plasmid using X‐tremeGENE HP DNA transfection reagents (Roche). Where indicated, 36 hours after transfection, human recombinant BMP6 (R&D systems) was added to the wells to a final concentration of 10 ng/mL. After incubating for an additional 12 hours, the cells were collected for the following analyses.

### 2.6 Western blot analysis

Cultured cells were lysed using RIPA lysis buffer, and total protein (40 μg/sample) was loaded on a 10% sodium dodecyl sulphate polyacrylamide gel. The following primary antibodies were used in this study: rabbit anti‐L‐ferritin (Alpha Diagnostics International), rabbit anti‐phosphor–Smad1/5/8 (Cell Signaling Technology), rabbit anti‐Smad1 antibody (Cell Signaling Technology), rabbit anti‐phosphor–Stat3 (Cell Signaling Technology), rabbit anti‐Stat3 (Cell Signaling Technology) and mouse anti‐β‐actin (Sigma‐Aldrich).

### 2.7 RNA extraction and real‐time PCR analysis

RNA extraction and real‐time PCR analysis of gene expression were performed as previously described.^26^ Relative expression was normalized to internal control β*‐actin*. The primer sequences are listed in Table S1.

### 2.8 RNA‐seq data analysis

Eight‐week‐old female *Smad7* ^*flox/flox*^ and *Smad7* ^*Alb/Alb*^ mice were fed an iron‐rich diet for 3 days. Total RNA was then isolated from the livers (3 mice per genotype), and RNA sequencing libraries were generated using the TruSeq RNA Sample Preparation Kit (Illumina). The Illumina HiSeq 2000 platform was used with 100‐bp paired‐end reads in accordance with the manufacturer's instructions. RNA‐seq reads were mapped to the mouse reference genome (mm9, NCBI build 37) using TopHat.^27^ Only uniquely aligned reads were used for gene and exon quantification. The Cufflinks tool was used to quantify isoform expression.^28^ Genes that were significantly up‐regulated (*q *<* *0.05) are listed in Table S2. These genes were then mapped to signalling pathways using the KEGG pathway mapping tool (http://www.genome.jp/kegg/tool/map_pathway1.html).

### 2.9 Statistical analysis

All summary data are presented as the mean ± SD. The Student's *t* test was used to compare two groups. For multiple group comparisons, we used an ANOVA followed by Tukey's post hoc test. If data did not meet the assumption of homogeneity of variance (Bartlett's test), log‐transformed values were used in ANOVA. Differences were considered significant if *P < *.05. Statistical analyses were performed using R (http://www.r-project.org).

## 3 RESULTS

### 3.1 Liver‐specific deletion of Smad7 caused increased hepcidin expression and iron deficiency

Smad7 interacts with the TGF‐β type I receptor via the MH2 domain, preventing phosphorylation of effector Smad proteins.^29^ To generate mice with hepatocyte‐specific Smad7 deletion, mice carrying the *Smad7* conditional knockout allele (*Smad7* ^*flox/flox*^)^21^ were backcrossed with wild‐type C57BL/6 mice at least seven generations and then crossed with albumin‐Cre (Alb) transgenic mice, yielding *Smad7* liver‐specific knockout mice in which the MH2 domain in exon 4 of *Smad7* is deleted. Heterozygous hepatocyte‐specific knockout mice (*Smad7* ^*WT/Alb*^) were used to generate *Smad7* ^*flox/flox*^ and *Smad7* ^*Alb/Alb*^ mice. Primary hepatocytes were isolated from *Smad7* ^*Alb/Alb*^ mouse livers and had a 98% reduction in *Smad7* expression (Figure S1).

**Figure 1:**
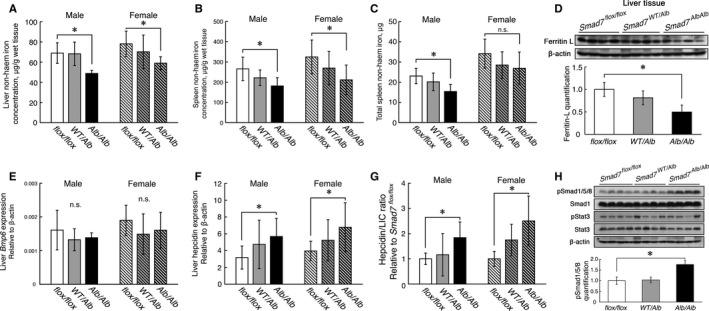
Hepatocyte‐specific *Smad7*‐knockout (*Smad7* ^*Alb/Alb*^) mice have increased liver hepcidin expression and develop an iron deficiency phenotype. Both male and female *Smad7* ^*Alb/Alb*^ mice have A, decreased liver; B, spleen tissue non‐haem iron concentrations; and C, total spleen iron (mice number: male, *Smad7* ^*flox/flox*^=8, *Smad7* ^*WT*^ ^*/Alb*^=8, *Smad7* ^*Alb/Alb*^=7; female, *Smad7* ^*flox/flox*^=20, *Smad7* ^*WT*^ ^*/Alb*^=9, *Smad7* ^*Alb/Alb*^=9). D, *Smad7* ^*Alb/Alb*^ mice have reduced levels of hepatic Ferritin‐L protein. E, *Smad7* ^*Alb/Alb*^ mice have unchanged *Bmp6* expression levels; F, increased levels of hepatic hepcidin (*Hamp1*) expression; and G, increased hepcidin expression/liver non‐haem iron concentration (hepcidin/LIC) ratio (mice number: male, *Smad7* ^*flox/flox*^=8, *Smad7* ^*WT*^ ^*/Alb*^=8, *Smad7* ^*Alb/Alb*^=7; female, *Smad7* ^*flox/flox*^=10, *Smad7* ^*WT*^ ^*/Alb*^=9, *Smad7* ^*Alb/Alb*^=9). H, *Smad7* ^*Alb/Alb*^ mice have increased levels of hepatic phosphor‐Smad1/5/8. *P < .05 vs *Smad7* ^*flox/flox*^ (ANOVA and Tukey's post hoc test; *P*‐values of hepcidin, *Bmp6*, hepcidin/LIC ratio were calculated from log‐transformed values)

Compared with control mice, both male and female *Smad7* ^*Alb/Alb*^ mice had reduced levels of non‐haem iron in the liver and spleen (Figure 1A‐C). *Smad7* ^*Alb/Alb*^ mice also had reduced levels of ferritin‐L protein in the liver, indicating decreased iron stores (Figure 1D). *Smad7* ^*Alb/Alb*^ mice showed no significant change in *Bmp6* expression (Figure 1E). Moreover, no difference was observed with respect to the ratio of *Bmp6* to liver non‐haem iron concentration (*Bmp6*/LIC ratio) (Figure S2) or *Tmprss6* expression (Figure S3). *Smad7* ^*Alb/Alb*^ mice had increased expression of hepcidin in the liver (Figure 1F) and an increased ratio of hepcidin to liver non‐haem iron concentration (hepcidin/LIC ratio) (Figure 1G). Consistently, *Smad7* ^*Alb/Alb*^ mice had increased levels of phosphor‐Smad1/5/8 (pSmad1/5/8) in the liver (Figure 1H).

**Figure 2:**
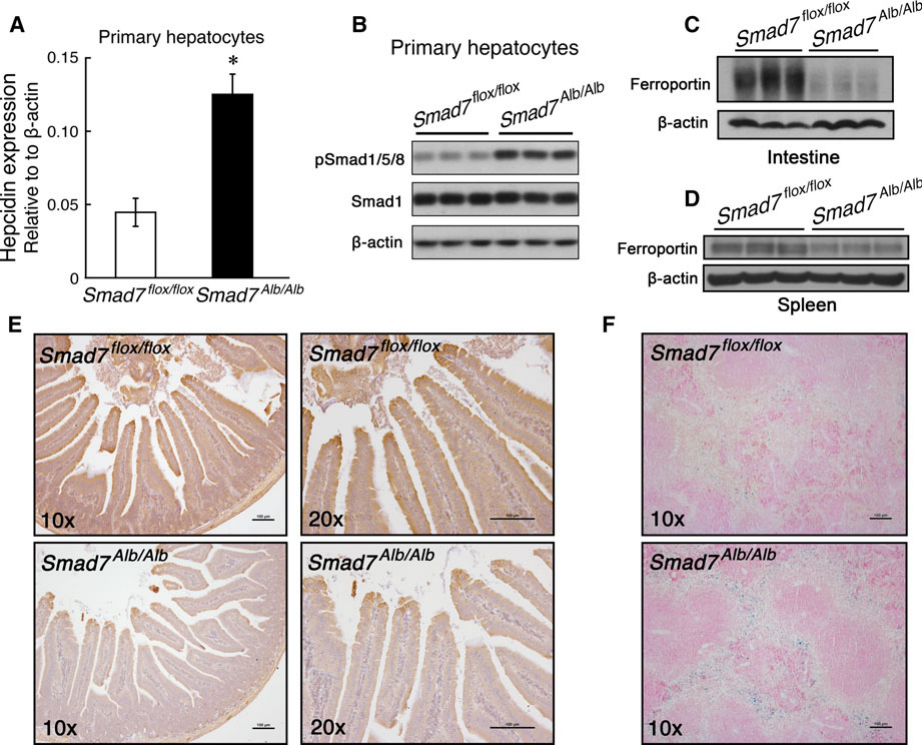
*Smad7* ^*Alb/Alb*^ mice have increased hepcidin expression in primary hepatocytes and decreased ferroportin protein levels in the intestine and spleen. A, Hepcidin (*Hamp1*) mRNA and B, Phosphor‐Smad1/5/8 levels in primary hepatocytes isolated from *Smad7* ^*flox/flox*^ and *Smad7* ^*Alb/Alb*^ mice (n = 3 mice/group; *P < .05, Student's *t* test). C, Intestinal and D, Splenic ferroportin protein was measured in *Smad7* ^*flox/flox*^ and *Smad7* ^*Alb/Alb*^ mice using Western blot analysis. E, Immunohistochemistry of ferroportin in the intestine tissues of *Smad7* ^*flox/flox*^ and *Smad7* ^*Alb/Alb*^ mice. F, Perls’ Prussian blue staining indicates iron retention in the spleen tissue of *Smad7* ^*Alb/Alb*^ mice

Isolated hepatocytes from *Smad7* ^*Alb/Alb*^ mice had significantly higher hepcidin expression and phosphor‐Smad1/5/8 levels (Figure 2A and B). Consistent with these results, *Smad7* ^*Alb/Alb*^ mice had decreased levels of ferroportin in the intestine and spleen (Figure 2C and D, respectively); decreased ferroportin in the intestines of *Smad7* ^*Alb/Alb*^ mice was confirmed using immunohistochemistry (Figure 2E). Perls’ Prussian blue staining indicates increased iron retention in the spleen (Figure 2F).

An analysis of serum samples revealed that *Smad7* ^*Alb/Alb*^ mice have decreased serum iron (SI) and transferrin saturation (TS) levels (Table 1). Moreover, *Smad7* ^*Alb/Alb*^ mice have an altered haematological profile, including decreased haemoglobin concentration, mean corpuscular volume (MCV), mean corpuscular haemoglobin (MCH) and mean corpuscular haemoglobin concentration (MCHC). Taken together, these serum and haematology results indicate that *Smad7* ^*Alb/Alb*^ mice have mild iron deficiency anaemia. The serum and haematology data are summarized in Table 1.

**Table 1:** Serum and haematological parameters of *Smad7* ^*flox/flox*^, *Smad7* ^*WT/Alb*^ and *Smad7* ^*Alb/Alb*^ mice

	*Smad7* ^*flox/flox*^		*Smad7* ^*WT/Alb*^		*Smad7* ^*Alb/Alb*^	
	Mean ± SD	n	Mean ± SD	n	Mean ± SD	n
Serum parameters						
Serum iron, μg/dL	106.34 ± 16.74	18	95.30 ± 19.36	7	76.86 ± 12.35^a^	7
UIBC, μg/dL	223.86 ± 44.97	18	296.33 ± 49.90^a^	7	302.18 ± 52.41^a^	7
TIBC, μg/dL	330.21 ± 38.78	18	387.04 ± 46.45^a^	7	379.04 ± 41.51^a^	7
TS, %	32.70 ± 6.60	18	23.80 ± 5.32^a^	7	20.72 ± 5.24^a^	7
Haematology						
Haemoglobin, g/L	133.67 ± 5.56	18	125.29 ± 4.72^a^	7	122.29 ± 8.92^a^	7
RBC	8.49 ± 0.43	18	8.30 ± 0.40	7	8.74 ± 1.16	7
Haematocrit, %	40.85 ± 2.41	18	39.96 ± 1.76	7	38.79 ± 3.45	7
MCV, fL	48.13 ± 1.24	18	48.14 ± 1.25	7	44.63 ± 2.76^a^	7
MCH, pg	15.76 ± 0.48	18	15.10 ± 0.46^a^	7	14.06 ± 0.62^a^	7
MCHC, g/L	327.72 ± 12.47	18	313.71 ± 9.29^a^	7	315.71 ± 10.40^a^	7

MCH, mean corpuscular haemoglobin; MCHC, mean corpuscular haemoglobin concentration; MCV, mean corpuscular volume; TIBC, total iron‐binding capacity; TS, transferrin saturation; UIBC, unsaturated iron‐binding capacity.

a *P *<* *.05, compared with *Smad7* ^*flox/flox*^ mice; *P*‐values were calculated using ANOVA and Tukey's post hoc test.

### 3.2 *Smad7^Alb/Alb^* mice fed an iron‐rich diet had up‐regulated expressions of *Smad6, Fst* and *Bambi*

The expression of *Smad7* has been linked to dietary iron.^19^, ^20^, ^30^ We thus have been suggested that feeding *Smad7* ^*Alb/Alb*^ mice with an iron‐rich diet might induce a more robust phenotype. According to the report, the hepcidin level reached to its peak at the 3rd day of iron‐rich diet treatment.^30^ We therefore choose a 3‐day iron‐rich diet treatment for the following experiments.

**Figure 3:**
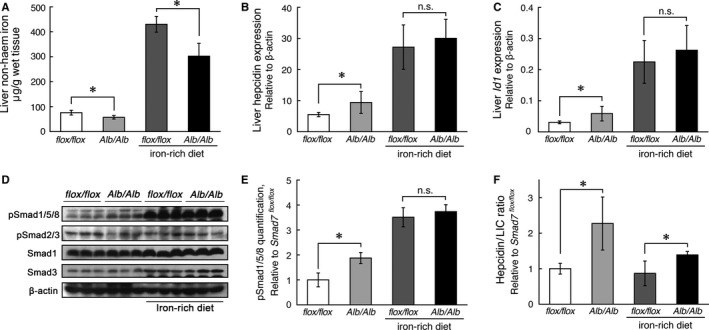
Hepcidin expression is similar between iron‐challenged *Smad7* ^*flox/flox*^ and iron‐challenged *Smad7* ^*Alb/Alb*^ mice. *Smad7* ^*flox/flox*^ and *Smad7* ^*Alb/Alb*^ mice were fed an iron‐rich diet for 3 days, after which A, liver non‐haem iron concentration; B, hepcidin; C, *Id1;* and D, phosphor‐Smad1/5/8 (pSmad1/5/8) and phosphor‐Smad2/3 (pSmad2/3) were measured. E, Quantification of hepatic phosphor‐Smad1/5/8 (pSmad1/5/8) using Western blot analysis. F, Summary of hepcidin expression/liver non‐haem iron concentration (hepcidin/LIC) ratio in *Smad7* ^*flox/flox*^ and *Smad7* ^*Alb/Alb*^ mice. *P < .05 vs the corresponding *Smad7* ^*flox/flox*^ (ANOVA and Tukey's post hoc test). In A‐C and E‐F, n = 5‐6 female mice/group

Although iron‐challenged *Smad7* ^*Alb/Alb*^ mice had considerably higher liver non‐haem iron concentrations compared with *Smad7* ^*Alb/Alb*^ *mice* that were fed a normal diet, these mice still had a lower liver non‐haem iron concentration compared with iron‐challenged *Smad7* ^*flox/flox*^ mice (Figure 3A). Interestingly, we found no difference in hepcidin expression, *Id1* expression or phosphor‐Smad1/5/8 levels between iron‐challenged *Smad7* ^*flox/flox*^ and iron‐challenged *Smad7* ^*Alb/Alb*^ mice (Figure 3B‐E). With respect to hepcidin/LIC ratio, consuming an iron‐rich diet reduced the difference between *Smad7* ^*Alb/Alb*^ and *Smad7* ^*Alb/Alb*^ mice (Figure 3F). These data suggest other factors could compensate Smad7's function in repressing hepcidin expression.

To identify these potential factors, we performed RNA‐seq analysis and examined which genes were differentially expressed in the liver between *Smad7* ^*Alb/Alb*^ mice and *Smad7* ^*flox/flox*^ mice under iron‐rich dietary condition. A total of 52 genes were significantly up‐regulated in *Smad7* ^*Alb/Alb*^ mice (*q *<* *0.05) and were selected for further analysis; these 52 genes are listed in Table S2. The genes were mapped to signalling pathways using the KEGG pathway mapping, and the pathways with ≥5 hits are summarized in Table 2; all pathways with ≥3 hits are summarized in Table S3.

**Table 2:** TGF‐β signalling pathway is one of the most hit pathways (hits ≥ 5) in all significantly up‐regulated genes in *Smad7* ^*Alb/Alb*^ mice liver under iron‐rich diet

Gene		Fold change	q value	Rank (by q value)^a^
Metabolic pathways				
*Pnpla3*	patatin‐like phospholipase domain containing 3	8.58	<2.54E‐11	1/6484
*Mvd*	mevalonate (diphospho) decarboxylase	3	8.16E‐05	14/6484
*Gck*	glucokinase	2.65	8.49E‐04	20/6484
*Acsl3*	acyl‐CoA synthetase long‐chain family member 3	2.58	7.03E‐03	35/6484
*Itpk1*	inositol 1,3,4‐triphosphate 5/6 kinase	3	7.35E‐03	36/6484
*Hmgcr*	3‐hydroxy‐3‐methylglutaryl‐Coenzyme A reductase	2.41	0.03	47/6484
TGF‐β signalling pathway				
*Smad6*	SMAD family member 6	4.42	1.04E‐08	4/6484
*Bambi*	BMP and activin membrane‐bound inhibitor	4.09	1.13E‐06	7/6484
*Id4*	inhibitor of DNA binding 4	6.6	8.60E‐06	10/6484
*Fst*	Follistatin	3.17	1.00E‐03	21/6484
*Id2*	inhibitor of DNA binding 2	2.41	5.83E‐03	32/6484
Longevity regulating pathway				
*Hspa1b*	heat‐shock protein 1B	4.09	1.00E‐07	5/6484
*Foxa2*	forkhead box A2	2.38	4.39E‐03	30/6484
*Adcy1*	adenylate cyclase 1	5.38	4.63E‐04	19/6484
*Hspa1a*	heat‐shock protein 1A	3.21	0.02	45/6484
*Hspa2*	heat‐shock protein 2	2.46	0.02	46/6484

q values of differentially expressed genes in RNA sequencing were calculated by Cufflink. Full list of significantly up‐regulated genes (q value < 0.05) was summarized in Table S2. Full list of pathways with hits ≥ 3 genes was summarized in Table S3. Genes were mapped into pathways using KEGG pathway mapping.

a in all up‐regulated genes.

Among the top‐rated pathways (Table 2), metabolic or longevity regulating pathways are not typical signal transduction pathways. Only the TGF‐β family members share similarities in both function and conservativeness across species. In addition, TGF‐beta family members are the most relevant molecules in regulating hepcidin. Particularly, the proteins encoded by the *Smad6*,* Bambi* and *Fst* genes are negative regulators of TGF‐β signalling. Bambi interacts with membrane BMP receptors to inhibit BMP signal transduction,^31^ and Smad6 inhibits the phosphorylation of Smad proteins. *Fst* encoding protein Follistatin binds to activin and BMPs, thereby blocking downstream signalling.^32^ Based on these functions, we investigated whether these proteins played a role in limiting iron‐induced hepcidin expression in the absence of Smad7.

### 3.3 Smad6, Bambi and Fst are differentially controlled by the iron‐BMP–Smad pathway

Because we found no detectable change in phosphor‐Smad2/3 levels (Figure 3D) or activin expression (Figure S4) in iron‐challenged *Smad7* ^*Alb/Alb*^ mice, we tested whether the BMP6‐Smad1/5/8 pathway controls these putative negative regulators of hepcidin. Accordingly, we measured the mRNA levels of *Smad6*,* Bambi* and *Fst* in *Hfe* ^*−/−*^ and *Smad7* ^*Alb/Alb*^ mice fed either a normal iron diet or an iron‐rich diet; we selected these two mouse lines because *Hfe* ^*−/−*^ mice have impaired BMP6‐Smad1/5/8 signalling, whereas *Smad7* ^*Alb/Alb*^ mice have enhanced signalling.

**Figure 4:**
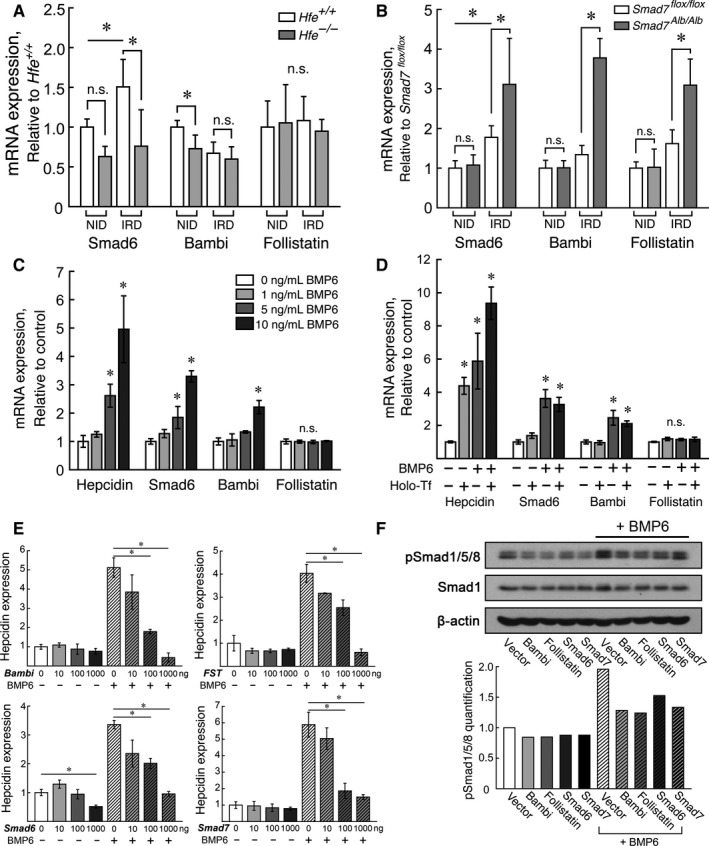
Smad6, Bambi and Follistatin are differentially controlled by the iron‐BMP–Smad pathway. Under either a normal iron diet (NID) or an iron‐rich diet (IRD), *Smad6*,* Bambi* and *Fst* (Follistatin) expressions were measured in A, male *Hfe* ^*−/−*^ mice and B, female *Smad7* ^*Alb/Alb*^ mice; n = 5‐6 mice/group. C, The expressions of hepcidin, *Smad6* and *Bambi*—but not *Fst* (Follistatin) —are up‐regulated in mouse primary hepatocytes after 10 ng/mL BMP6 treatment for 6 h (n = 3 replicates/group). D, Hepcidin, *Smad6*,* Bambi* and *Fst* (Follistatin) expressions were measured in primary hepatocytes treated with 10 ng/mL BMP6 and/or 50 μmol/L holo‐Transferrin for 12 h (n = 3 replicates/group). E‐F, Huh7 cells were transfected with 0, 10, 100 or 1000 ng of plasmid encoding Smad6, Smad7, Bambi or Follistatin; 36 h after transfection, the cells were incubated for 12 h in the absence or presence of 10 ng/mL BMP6, after which (E) hepcidin expression and (F) phosphor‐Smad1/5/8 (pSmad1/5/8) levels were measured. In (F), the cells were transfected with 1000 ng of the indicated plasmid. β‐actin was used as an internal control, and the results are presented relative to control vector‐transfected, untreated cells. *P < .05 (ANOVA and Tukey's post hoc test)

We found decreased hepatic expression of *Smad6* in *Hfe* ^*−/−*^ mice (Figure 4A) and increased hepatic expression of *Smad6* in *Smad7* ^*Alb/Alb*^ mice (Figure 4B). Moreover, *Smad6* expression changed in response to an iron‐rich diet (Figure 4A and B) and BMP6 treatment (Figure 4C) in mice and primary hepatocytes, respectively. Similarly, *Bambi* expression decreased slightly in *Hfe* ^*−/−*^ mice but increased in iron‐challenged *Smad7* ^*Alb/Alb*^ mice (Figure 4A and B). *Bambi* expression also increased in primary hepatocytes in response to 10 ng/mL BMP6 (Figure 4C), which indicates that Bambi is also regulated by the BMP6‐Smad1/5/8 pathway. In contrast, the expression of *Fst* (Follistatin) was not affected by either an iron‐rich diet or BMP6 treatment (Figure 4A‐C), with the exception of increased *Fst* expression in *Smad7* ^*Alb/Alb*^ mice fed an iron‐rich diet (Figure 4B); this finding suggests that up‐regulation of Follistatin only occurs at *Smad7*‐deficient condition. Treating primary hepatocytes with holo‐Transferrin had no effect on *Smad6, Bambi or Fst* expressions (Figure 4D), and overexpressing Smad7 had no effect on *Smad6, Bambi* or *Fst* expressions either (Figure S5). These results indicate that only Smad6 and Bambi are regulated by the BMP6‐Smad1/5/8 pathway.

### 3.4 Overexpression of *Smad6, Bambi* or *Fst* decreases hepcidin expression

**Figure 5:**
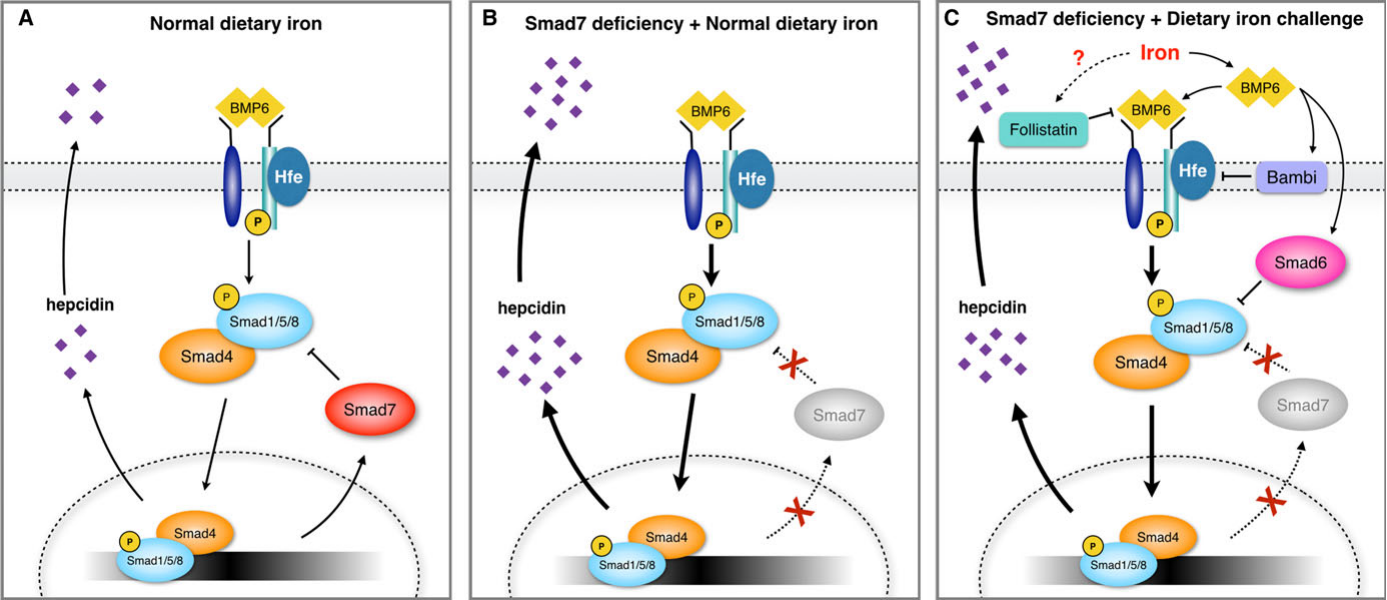
Proposed model describing the regulatory pathway between Smad7 and hepcidin expression in hepatocytes. A, Under normal conditions, hepcidin expression is regulated by BMP6 signalling via a complex comprised of Smad4 and phosphor‐Smad1/5/8. Smad7 inhibits hepcidin expression by targeting the Smad4‐phosphor‐Smad1/5/8 complex. B, Deletion of Smad7 in hepatocytes leads to increased hepcidin expression. C, During dietary iron overload, Smad6, Bambi and Follistatin assume the inhibitory function of Smad7, thereby functionally taking the place of Smad7 in inhibiting BMP‐Smad signalling

To further investigate whether Smad6, Bambi and/or Follistatin are negative regulators of hepcidin expression, we overexpressed Smad6, Smad7, Bambi and Follistatin in Huh7 cells. Forty‐eight hours after transfection, hepcidin mRNA and phosphor‐Smad1/5/8 levels were decreased compared with control‐transfected cells (Figure 4E and F). Moreover, overexpressing either Bambi or Follistatin abrogated BMP6‐induced hepcidin expression (Figure 4E). These findings suggest that under normal iron conditions, these inhibitory factors cannot fully replace the inhibitory function of Smad7; however, when the system is challenged by an iron‐rich diet, Bambi, Follistatin and Smad6 can assume the inhibitory role of Smad7 in regulating hepcidin expression (Figure 5).

## 4 DISCUSSION

Hepcidin, a liver‐derived antimicrobial peptide, is a key regulator of iron homoeostasis and anaemia of inflammation. At the mechanistic level, intracellular iron sequestration is mediated by the hepcidin‐induced internalization and degradation of ferroportin, the only iron exporter identified to date.^3^ The hepcidin‐ferroportin axis is therefore a promising therapeutic target for treating many iron disorders, including haemochromatosis, β‐thalassaemia, anaemia of chronic disease and iron‐refractory iron deficiency anaemia.^9^

Hepcidin expression is tightly regulated by the BMP‐Smad signalling pathway. Although Smad7 has been suggested to regulate hepcidin expression based on in vitro studies*,* 18, 19 this function has not been investigated in vivo. In cell lines, overexpressing Smad7 reduced hepcidin expression.^18^ Thus, Smad7 may serve as a negative feedback regulator of hepcidin expression. Global *Smad7*‐knockout (*Smad7* ^*−/−*^) mice have reduced viability,^21^, ^33^ impaired cardiovascular development,^21^ exacerbated liver injury^34^ and altered immune cell responses.^35^ To investigate the physiological role of Smad7 in regulating both hepcidin expression and iron metabolism, we generated hepatocyte‐specific *Smad7*‐knockout (*Smad7* ^*Alb/Alb*^) mice. Consistent with a previous study by Zhu et al,^36^ a small subset of *Smad7* ^*Alb/Alb*^ mice are slightly smaller in size compared with control mice. Zhu et al reported that 30% of *Smad7* ^*Alb/Alb*^ mice displayed spontaneous liver dysfunction and increased serum levels of AST and ALT. However, we did not observe any liver damage as measured by serum AST and ALT (Figure S6). The difference between 2 studies could be attributed to several aspects. First, the genetic background differs. In our study, *Smad7* ^*Alb/Alb*^ mice were backcrossed with C57BL/6 for at least 7 generations, whereas Zhu et al used a mixed background without any backcrossing. Second, the age of the mice differs. We used 8‐week‐old mice, whereas they used 10~12‐week‐old mice.

Both liver tissue and primary hepatocytes obtained from *Smad7* ^*Alb/Alb*^ mice have increased hepcidin expression and increased phosphor‐Smad1/5/8 levels, which is consistent with increased phosphor‐Smad1/5/8 levels reported in other cell types obtained from *Smad7* ^*−/−*^ mice.^37^ Increased hepcidin expression in *Smad7* ^*Alb/Alb*^ mice leads to impaired iron metabolism, including decreased serum iron levels, decreased transferrin saturation and decreased tissue non‐haem iron concentration. Moreover, we found decreased levels of ferroportin protein in the intestine of *Smad7* ^*Alb/Alb*^ mice, which suggests impaired absorption of dietary iron, thereby explaining the iron deficiency phenotypes present in *Smad7* ^*Alb/Alb*^ mice. Interestingly, we also observed a slight reduction in total splenic ferroportin protein levels. Although reduced splenic ferroportin levels will cause iron retention in splenic macrophages (Figure 2F), splenic non‐haem iron concentration was still lower in the *Smad7* ^*Alb/Alb*^ mice. Thus, we conclude that the slight reduction in splenic ferroportin cannot fully counteract the systemic iron deficiency in *Smad7* ^*Alb/Alb*^ mice. Moreover, we measured reduced haematological parameters in both homozygous and heterozygous hepatocyte‐specific knockout mice. Heterozygotes (*Smad7* ^*WT/Alb*^) mice displayed significant reductions in TIBC, haemoglobin, MCH and MCHC compared with *Smad7* ^*flox/flox*^ mice. However, tissue iron concentrations and hepatic hepcidin expression remain unchanged, which suggest that blood iron parameters are more sensitive to iron deficiency. Similarly, deleting *Tmprss6*, which encodes a serine protease that represses hepcidin expression, causes decreased liver non‐haem iron concentration and decreased mean corpuscular volume; however, *Tmprss6* knockout mice developed a more severe phenotype than *Smad7* ^*Alb/Alb*^ mice.^38^ In addition, a recent study suggested that Tmprss6 plays a key role in erythroferrone‐mediated hepcidin suppression.^39^ In contrast, hepatic expression of *Tmprss6* remains unchanged in our *Smad7* ^*Alb/Alb*^ mice (Figure S3). Taken together, these data indicate that Smad7 together with other hepcidin‐negative regulators plays an essential role in maintaining iron homoeostasis.

*Smad7* expression is up‐regulated in mice fed an iron‐rich diet and down‐regulated in mice fed an iron‐deficient diet.^20^ Given its role as a negative regulator of hepcidin, we have been suggested that deleting *Smad7* would increase hepcidin's response to dietary iron. Interestingly, however, we found no difference in hepcidin up‐regulation between *Smad7* ^*Alb/Alb*^ and *Smad7* ^*flox/flox*^ mice after 3 days on an iron‐rich diet; phosphor‐Smad1/5/8 and phosphor‐Smad2/3 levels were not affected, either. On the other hand, our RNA‐seq analysis revealed that Smad6, Bambi and Follistatin are inhibitory factors of the TGF‐β signalling pathway.

Our finding indicates that deleting either *Hfe* or *Smad4* significantly reduced *Smad6* expression and abolished the iron‐induced increase in *Smad6* expression (Figure 4A and Figure S7), which supports the notion that Smad6 is a downstream target of BMP‐Smad signalling. In addition, we found that *Smad6* expression was unchanged in *Smad7* ^*Alb/Alb*^ mice compared with wild‐type mice, which indicates that Smad6 cannot fully replace the role of Smad7 in the BMP‐Smad–hepcidin axis, even though both Smad6 and Smad7 are well‐characterized inhibitory Smads. This notion is supported by the non‐overlapping phenotypes between *Smad7*‐knockout mice and *Smad6*‐knockout mice.^21^ Our in vivo data therefore suggest that inhibitory Smads have non‐redundant functions.

The bone morphogenetic protein Bambi exerts its inhibitory effect by interacting with TGF‐β type I receptors (including BMP receptors), thereby preventing the formation of the receptor complex and downstream phosphorylation of Smad proteins.^31^ We found that overexpressing Bambi inhibited Smad1/5/8 phosphorylation and reduces hepcidin expression. The promotor in the *Bambi* gene contains a BMP‐responsive element,^40^ and 10 ng/mL BMP6 significantly up‐regulated *Bambi* expression. In contrast, although iron is believed to up‐regulate hepatic *Bmp6* expression, we found no change in *Bambi* expression in response to an iron‐rich diet in our mouse models. This discrepancy may be explained by the different conditions used; dietary iron causes a relatively mild (2.5‐fold) increase in BMP6, whereas treating cultured hepatocytes with 10 ng/mL BMP6 represents a 20‐fold increase in BMP6 concentration.^41^

Follistatin can bind directly to BMPs, thereby inhibiting a variety of functions.^32^ Unlike Bambi, Follistatin expression did not respond to BMP6 treatment in primary hepatocytes. Therefore, up‐regulation of Follistatin expression requires additional, currently unknown physiological changes induced in the liver by dietary iron. One possible candidate is Nrf2, a transcription factor that is activated during oxidative stress and can direct bind to the *Fst* promoter to drive Follistatin expression.^42^ Iron deposition can induce oxidative stress and the nuclear translocation of Nrf2.^43^ It is therefore possible that the combined effects of deleting Smad7 and activating Nrf2 lead to Follistatin expression, which may also explain the slight increase in hepatic Follistatin levels in *Smad4* ^*Alb/Alb*^ mice. Smad4 mediates the transcription of *Smad7*, and deleting *Smad4* reduces *Smad7* expression.^44^, ^45^ Thus, the increased levels of hepatic Follistatin in *Smad4* ^*Alb/Alb*^ mice may have been caused by the combination of high iron deposition and reduced Smad7 expression.

Our data suggest that Smad6, Bambi and Follistatin collectively form a negative feedback circuit to suppress hepcidin expression via BMP‐Smad1/5/8 phosphorylation. Consistent with this notion, overexpressing Smad6, Bambi or Follistatin in Huh7 cells suppressed hepcidin expression, even in the presence of BMP6. However, Smad6, Bambi and Follistatin exert their inhibitory effects in different cellular compartments—Follistatin inhibits extracellular BMP ligand activity, Bambi inhibits the BMP receptor at the cell membrane, and Smad6 inhibits the phosphorylation of intracellular Smad proteins (see Figure 5).

In conclusion, we report that hepatic Smad7 plays an essential role in maintaining iron homoeostasis by negatively regulating the expression of hepcidin under basal (ie, normal dietary iron) conditions. When Smad7 is absent, Smad6, Bambi and Follistatin can take over the role of regulating hepcidin induction during high iron conditions by inhibiting BMP‐Smad1/5/8 signalling. The identification of Smad6, Bambi and Follistatin as novel‐negative regulators of hepcidin expression may have clinical implications regarding the treatment of iron‐related disorders.

## CONFLICT OF INTEREST

The authors confirm that there are no conflict of interests.

## AUTHOR CONTRIBUTIONS

FW and JM designed the study. YC provided mice with *Smad7* conditional allele. PA, HW, QW, JW, ZX, XW and XH performed the animal experiments. HW, PA, QW, XW and XH performed cell experiments. PA, HW and ZX performed data analyses. PA, HW, JM and FW drafted the manuscript.
