# Supplementary figures and images for: Smad7 deficiency decreases iron and haemoglobin through hepcidin up‐regulation by multilayer compensatory mechanisms
Source: J Cell Mol Med. 2018 Mar 25;22(6):3035–44. doi: 10.1111/jcmm.13546 (PMC5980186; doi:10.1111/jcmm.13546)

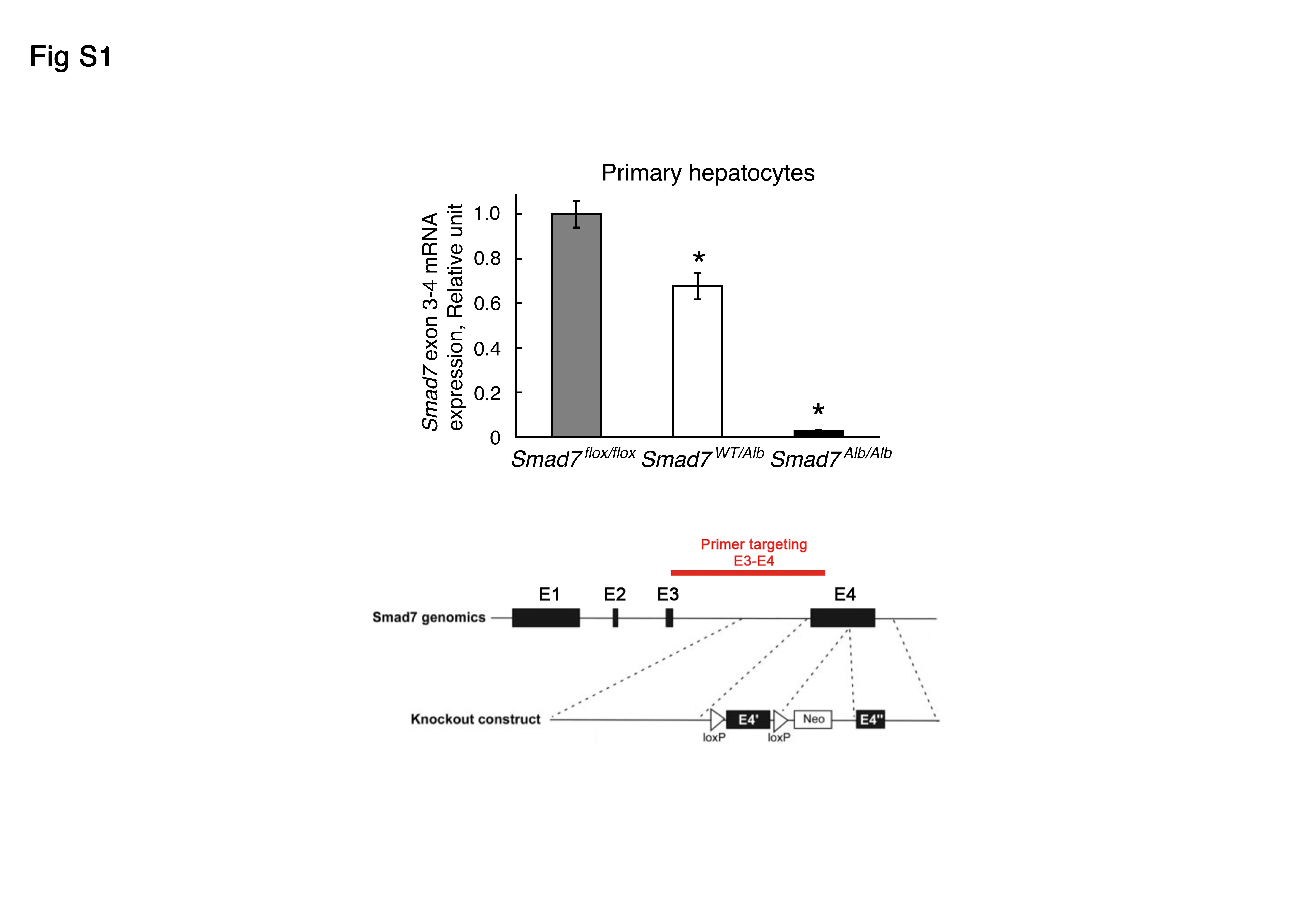

Supplement: Supplementary file 1 [file JCMM-22-3035-s001.tif]

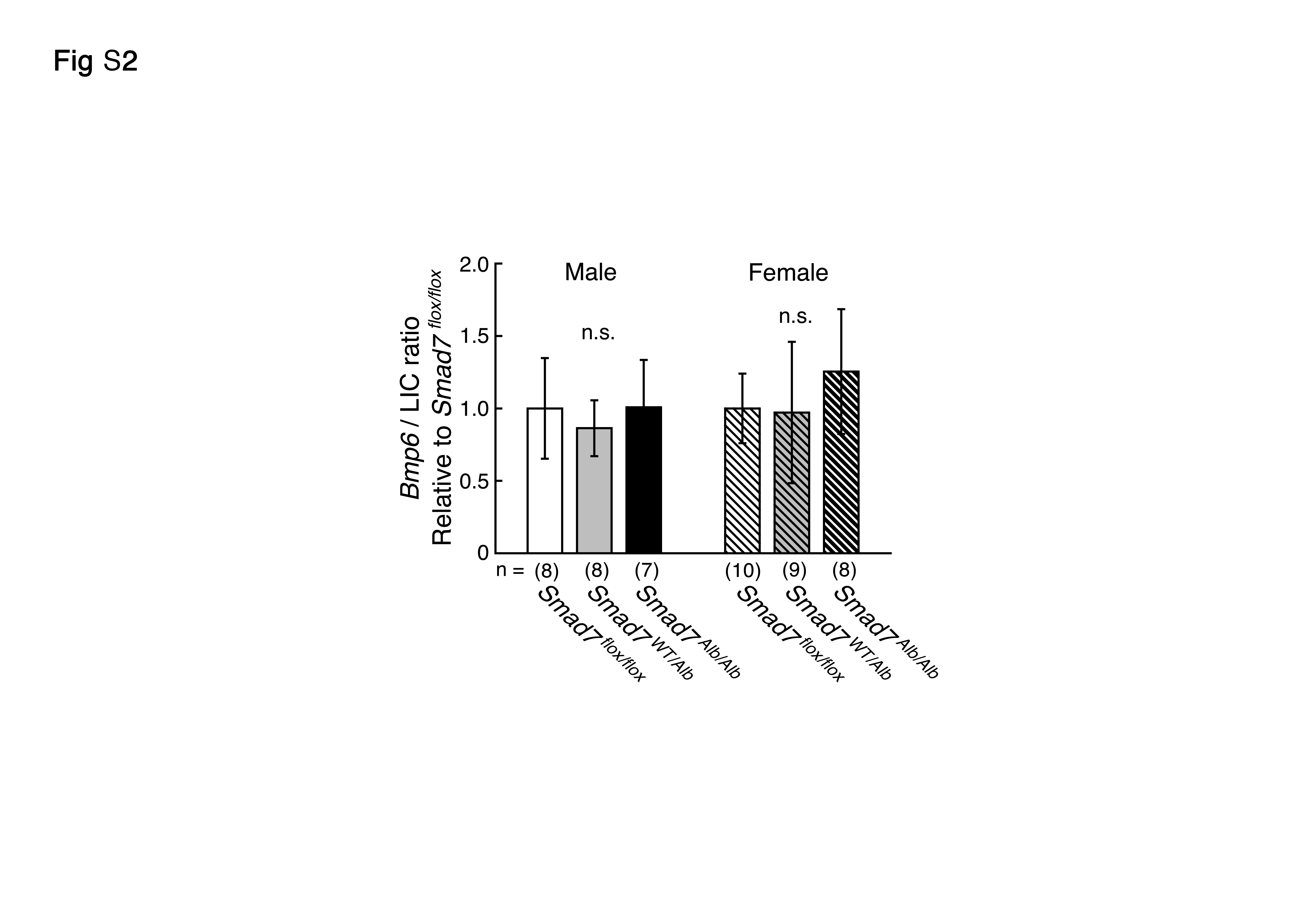

Supplement: Supplementary file 2 [file JCMM-22-3035-s002.tif]

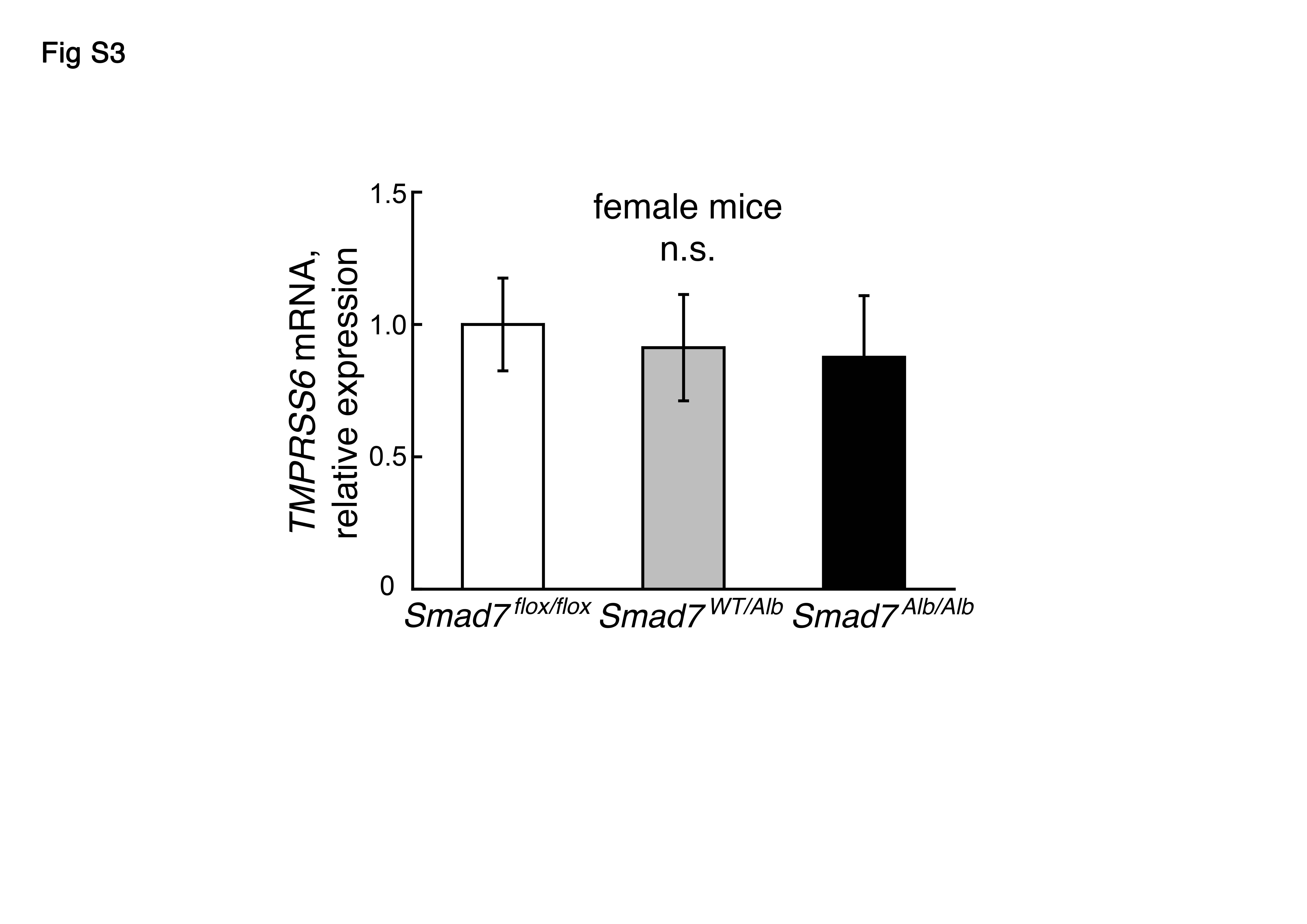

Supplement: Supplementary file 3 [file JCMM-22-3035-s003.tif]

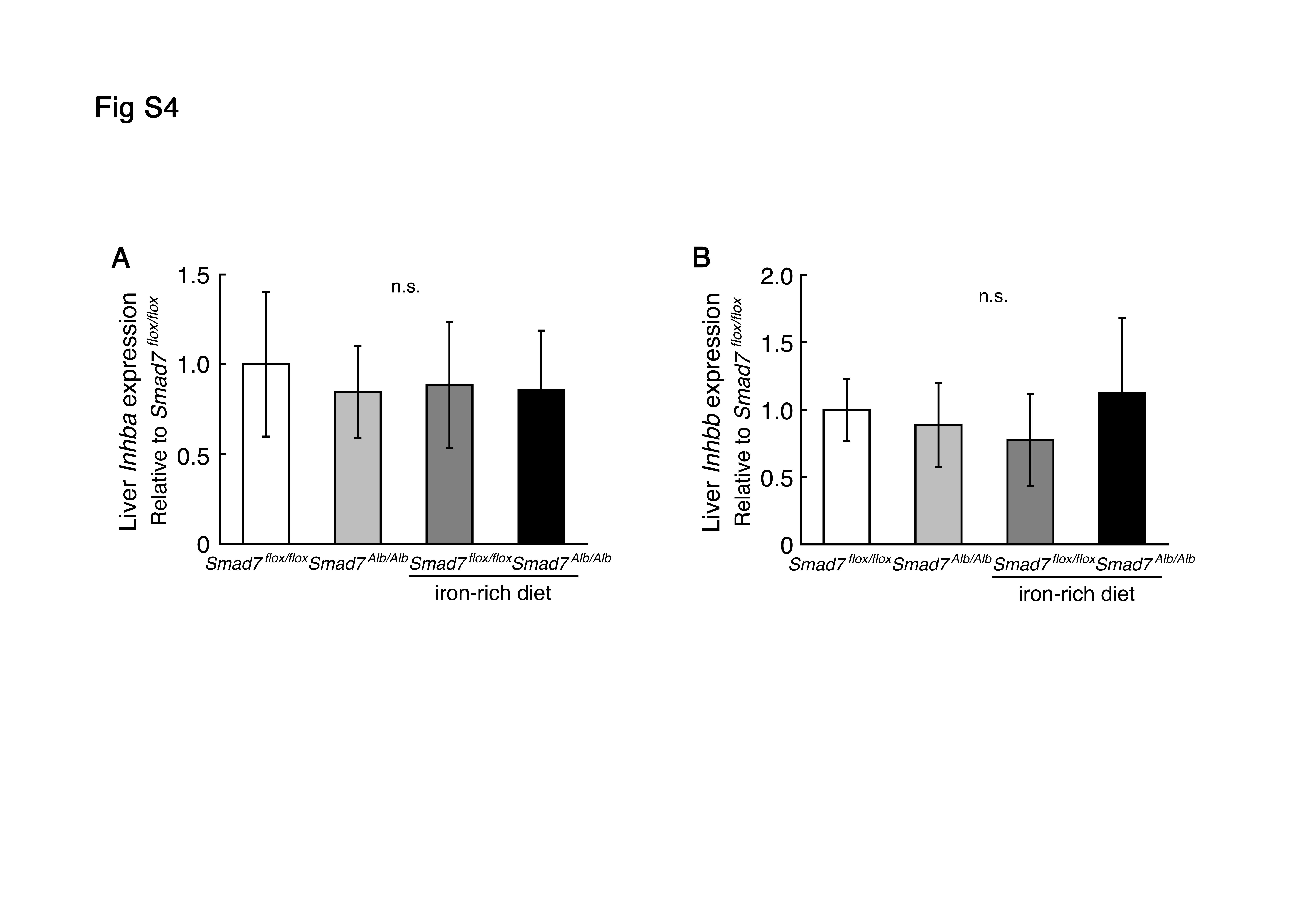

Supplement: Supplementary file 4 [file JCMM-22-3035-s004.tif]

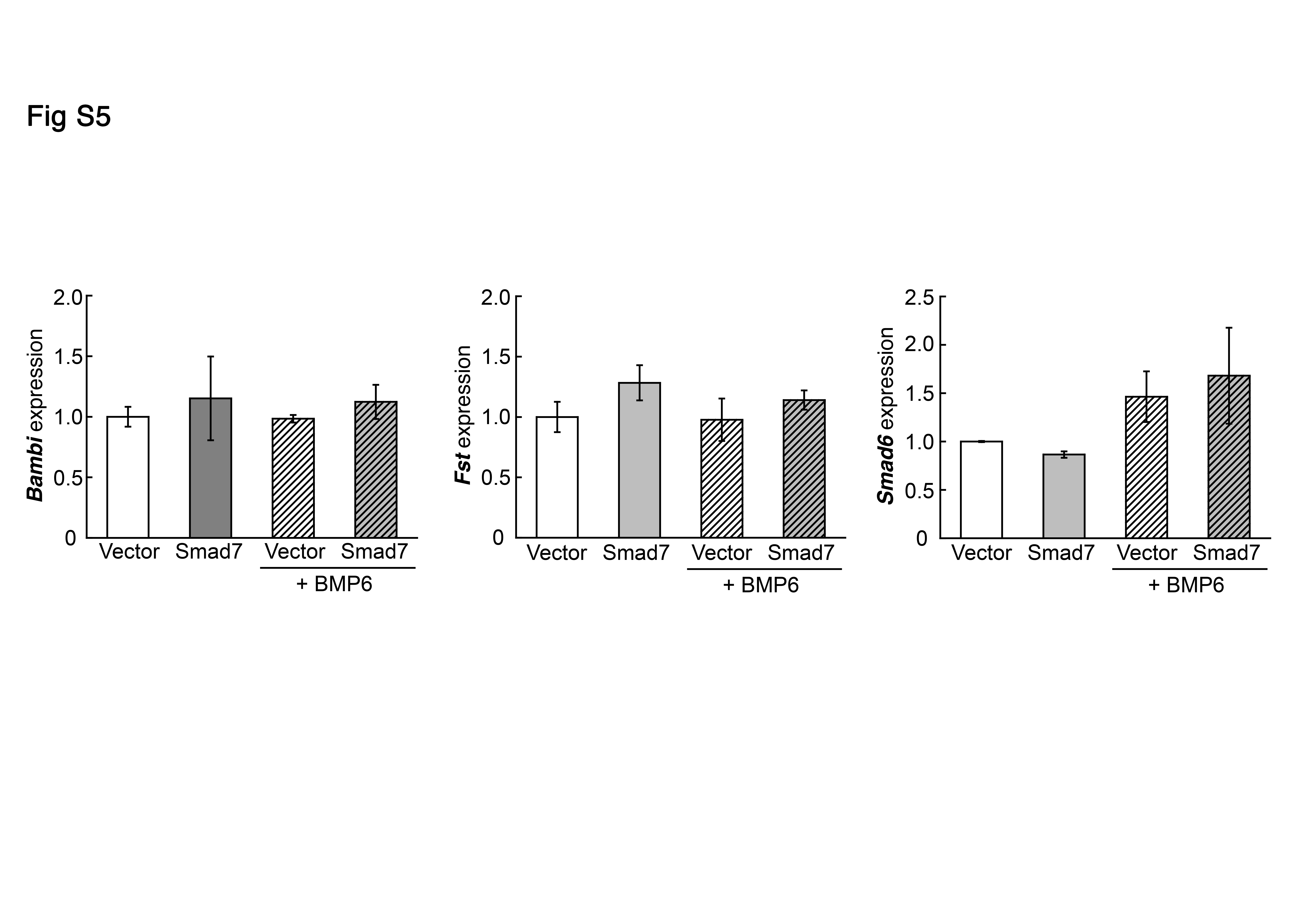

Supplement: Supplementary file 5 [file JCMM-22-3035-s005.tif]

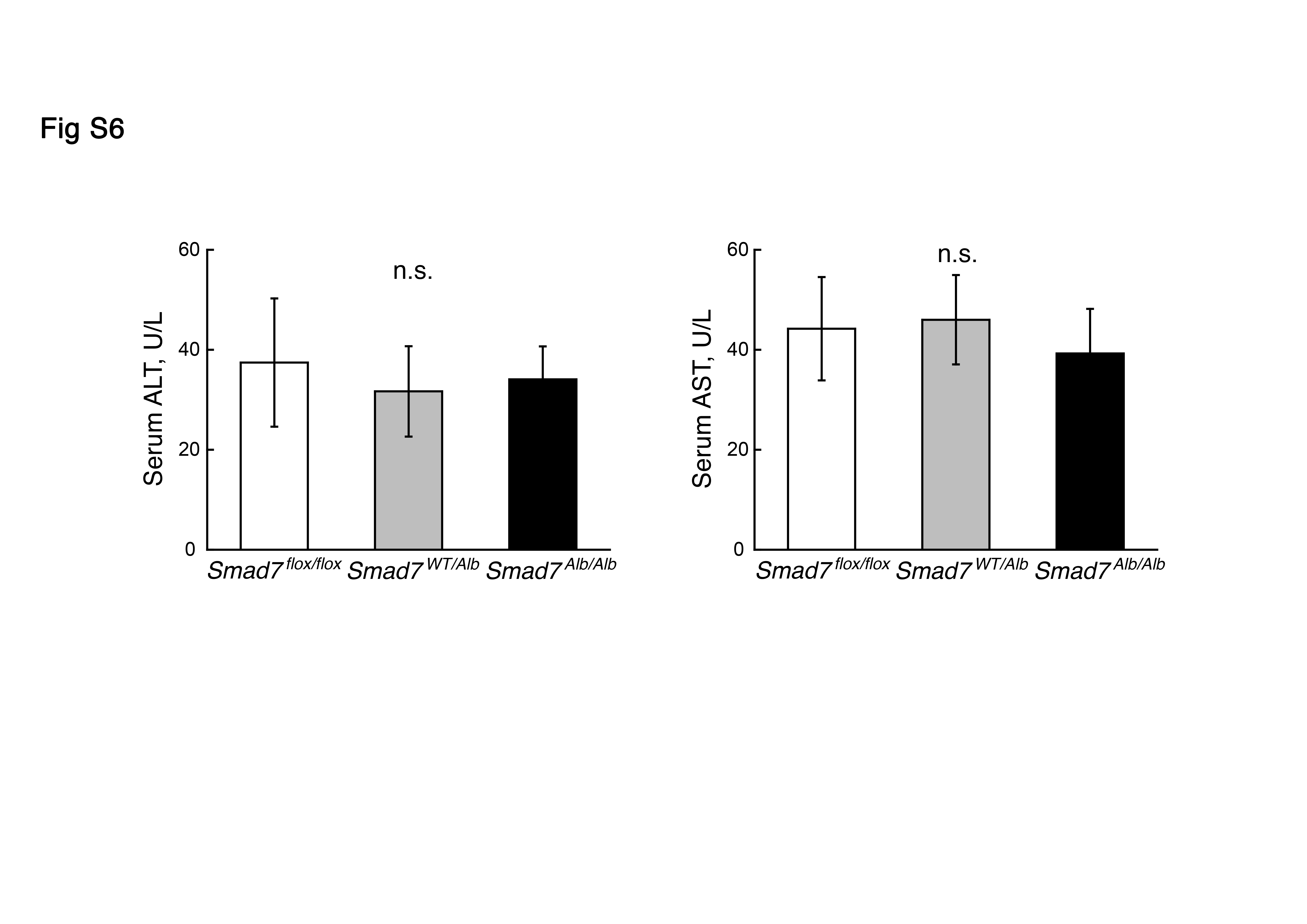

Supplement: Supplementary file 6 [file JCMM-22-3035-s006.tif]

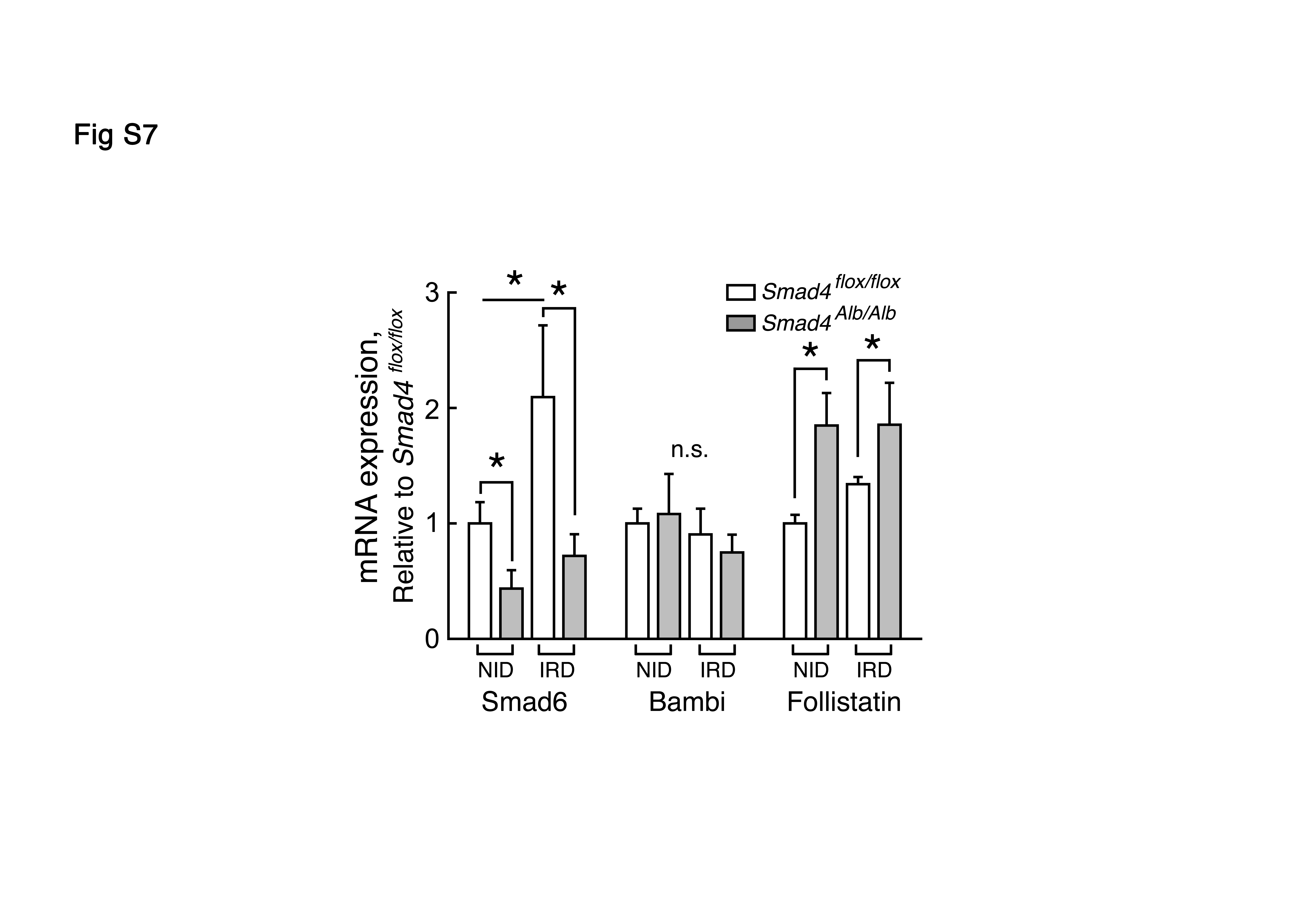

Supplement: Supplementary file 7 [file JCMM-22-3035-s007.tif]
