# Supplementary material for: Smad7 deficiency decreases iron and haemoglobin through hepcidin up‐regulation by multilayer compensatory mechanisms
Source: J Cell Mol Med. 2018 Mar 25;22(6):3035–44. doi: 10.1111/jcmm.13546 (PMC5980186; doi:10.1111/jcmm.13546)
Supplement: Supplementary file 8 [file JCMM-22-3035-s008.docx]

**Table S1.** Sequences of primers used for qPCR.

| **Gene** | **Forward** | **Reverse** |
| --- | --- | --- |
| *Smad7* (exon 1-3) | CTCCTGCTGTGCAAAGTGTT | GATTCACAGCAACACAGCCT |
| *Smad7* (exon 3-4) | TTCGGACAACAAGAGTCAGC | GGTAACTGCTGCGGTTGTAA |
| *Hamp1* | GCACCACCTATCTCCATCAACA | TTCTTCCCCGTGCAAAGG |
| *Bmp6* | ATGGCAGGACTGGATCATTGC | CCATCACAGTAGTTGGCAGCG |
| *Id1* | CGCAGCCACCGGACTCT | AACCCCCTCCCCAAAGTCT |
| *Tmprss6* | TTGCTGGTCTTGGCTGCGCT | AATGACGGTTGAGCACCCGGAG |
| *Smad6* | GTTGCAACCCCTACCACTTC | GGAGGAGACAGCCGAGAATA |
| *Fst* | TGGACCGAGGAGGATGTGA | CGCCCCCGTTGAAAATC |
| *Bambi* | CAGCAGCAGAAACCTCATCACT | GCAATGGGAACCGCTATCAC |
| *Actb* | AAATCGTGCGTGACATCAAAGA | GCCATCTCCTGCTCGAAGTC |
| *HAMP* | CAGCTGGATGCCCATGTTC | CAGCAGCCGCAGCAGAA |
| *ID1* | CTGGACGAGCAGCAGGTAAAC | GAATCTCCACCTTGCTCACCTT |
| *ACTB* | CACGGCATCGTCACCAACT | CACGCAGCTCATTGTAGAAGGT |

**Table S2.** List of genes that were significantly upregulated in RNA-seq using liver tissue from *Smad7^flox/flox^* and *Smad7^Alb/Alb^* mice under 3-day iron-rich diet**.**

| **Gene** | **fold change** | **q value ^A^** |
| --- | --- | --- |
| Pnpla3 | 8.579767092 | <2.54E-11 |
| Mid1ip1 | 5.287020387 | 2.66E-10 |
| Ihh | 9.282100956 | 8.21E-09 |
| Smad6 | 4.415206591 | 1.04E-08 |
| Hspa1b | 4.094192648 | 1.00E-07 |
| Hr | 8.876342277 | 1.89E-07 |
| Bambi | 4.093234244 | 1.13E-06 |
| Atp2b2 | 3.706535376 | 2.29E-06 |
| Serpina4-ps1 | 6.088420474 | 7.12E-06 |
| Id4 | 6.595048474 | 8.60E-06 |
| Slc2a4 | 4.505247401 | 1.04E-05 |
| H1fx | 10.12972147 | 1.14E-05 |
| Gm6484 | 4.235606344 | 1.22E-05 |
| Mvd | 2.995195589 | 8.16E-05 |
| Cib3 | 3.551379414 | 9.71E-05 |
| Slc13a3 | 2.771801164 | 0.000159926 |
| Cldn1 | 2.656195319 | 0.000398246 |
| Cd63 | 2.657081189 | 0.000442533 |
| Adcy1 | 5.379291925 | 0.00046298 |
| Gck | 2.64743733 | 0.00084895 |
| Fst | 3.166266498 | 0.00100249 |
| Adora1 | 2.53227807 | 0.00153786 |
| Bcl3 | 2.754806407 | 0.00156541 |
| Tmem50b | 2.606152032 | 0.00180704 |
| Enho | 2.758700793 | 0.00247469 |
| Spsb1 | 3.769839793 | 0.00272366 |
| 4930412O13Rik | 6.174138418 | 0.00364656 |
| 6330416G13Rik | 2.357283684 | 0.00376434 |
| Cyr61 | 2.563232239 | 0.00425892 |
| Foxa2 | 2.377441076 | 0.00439153 |
| Ppp1r3b | 2.911799702 | 0.00541589 |
| Tmem200b | 3.021433002 | 0.00583328 |
| Id2 | 2.414080351 | 0.00583328 |
| Nudt18 | 2.279173749 | 0.00694151 |
| Acsl3 | 2.581585266 | 0.00703402 |
| Itpk1 | 2.2652996 | 0.0073485 |
| Aacs | 2.665861546 | 0.00798936 |
| Gse1 | 2.486203641 | 0.00798936 |
| Grhl1 | 3.391095237 | 0.0104196 |
| Abtb2 | 2.473271645 | 0.0148102 |
| Slc22a7 | 2.346475499 | 0.0148102 |
| Hsph1 | 2.170481618 | 0.0177198 |
| Il1b | 3.781896258 | 0.0180134 |
| Cdh1 | 2.117805879 | 0.0214772 |
| Hspa1a | 3.205741562 | 0.0218791 |
| Hspa2 | 2.462584984 | 0.0218791 |
| Hmgcr | 2.405776759 | 0.0321582 |
| Pde4b | 2.647289913 | 0.0334819 |
| Rassf7 | 2.062522304 | 0.034836 |
| Rassf6 | 2.305589105 | 0.0354638 |
| H19 | 2.287629879 | 0.0383869 |
| Nkd1 | 2.319276944 | 0.0432214 |

^A^ q values are calculated using Cufflink.

**Table S3.** Pathway mapping of significantly upregulated genes in *Smad7^Alb/Alb^* mice liver under iron-rich diet (hits ≥ 3).

| **Gene** | | **fold change** | **q value** | **rank (by q value)*** |
| --- | --- | --- | --- | --- |
|  |  |  |  |  |
| **Metabolic pathways** | |  |  |  |
| *Pnpla3* | patatin-like phospholipase domain containing 3 | 8.58 | <2.54E-11 | 1/6484 |
| *Mvd* | mevalonate (diphospho) decarboxylase | 3 | 8.16E-05 | 14/6484 |
| *Gck* | glucokinase | 2.65 | 8.49E-04 | 20/6484 |
| *Acsl3* | acyl-CoA synthetase long-chain family member 3 | 2.58 | 7.03E-03 | 35/6484 |
| *Itpk1* | inositol 1,3,4-triphosphate 5/6 kinase | 3 | 7.35E-03 | 36/6484 |
| *Hmgcr* | 3-hydroxy-3-methylglutaryl-Coenzyme A reductase | 2.41 | 0.03 | 47/6484 |
|  |  |  |  |  |
| **TGF-β signaling pathway** | |  |  |  |
| *Smad6* | SMAD family member 6 | 4.42 | 1.04E-08 | 4/6484 |
| *Bambi* | BMP and activin membrane-bound inhibitor | 4.09 | 1.13E-06 | 7/6484 |
| *Id4* | inhibitor of DNA binding 4 | 6.6 | 8.60E-06 | 10/6484 |
| *Fst* | follistatin | 3.17 | 1.00E-03 | 21/6484 |
| *Id2* | inhibitor of DNA binding 2 | 2.41 | 5.83E-03 | 32/6484 |
|  |  |  |  |  |
| **Longevity regulating pathway** | |  |  |  |
| *Hspa1b* | heat shock protein 1B | 4.09 | 1.00E-07 | 5/6484 |
| *Foxa2* | forkhead box A2 | 2.38 | 4.39E-03 | 30/6484 |
| *Adcy1* | adenylate cyclase 1 | 5.38 | 4.63E-04 | 19/6484 |
| *Hspa1a* | heat shock protein 1A | 3.21 | 0.02 | 45/6484 |
| *Hspa2* | heat shock protein 2 | 2.46 | 0.02 | 46/6484 |
|  |  |  |  |  |
| **cAMP signaling pathway** | |  |  |  |
| *Adora1* | adenosine A1 receptor | 2.53227807 | 0.00153786 | 22/6484 |
| *Atp2b2* | ATPase, Ca++ transporting, plasma membrane 2 | 3.706535376 | 2.29E-06 | 8/6484 |
| *Pde4b* | phosphodiesterase 4B, cAMP specific | 2.647289913 | 0.033482 | 48/6484 |
| *Adcy1* | adenylate cyclase 1 | 5.379291925 | 4.63E-04 | 19/6484 |
|  |  |  |  |  |
| **Hippo signaling pathway** | |  |  |  |
| *Nkd1* | naked cuticle 1 homolog | 2.319276944 | 0.0432214 | 8/6484 |
| *Id2* | inhibitor of DNA binding 2 | 2.414080351 | 0.00583328 | 32/6484 |
| *Cdh1* | cadherin 1 | 2.117805879 | 0.021477 | 44/6484 |
| *Rassf6* | Ras association (RalGDS/AF-6) domain family member 6 | 2.305589105 | 0.0354638 | 50/6484 |
|  |  |  |  |  |
| **MAPK signaling pathway/ Legionellosis/ Influenza A/ Measles** | |  |  |  |
| *Hspa1b* | heat shock protein 1B | 4.094192648 | 1.00E-07 | 5/6484 |
| *Il1b* | interleukin 1 beta | 3.781896258 | 0.018013 | 43/6484 |
| *Hspa1a* | heat shock protein 1A | 3.205741562 | 0.021879 | 45/6484 |
| *Hspa2* | heat shock protein 2 | 2.462584984 | 0.0218791 | 46/6484 |
|  |  |  |  |  |
| **Protein processing in endoplasmic reticulum** | |  |  |  |
| *Hspa1b* | heat shock protein 1B | 4.094192648 | 1.00E-07 | 5/6484 |
| *Hsph1* | heat shock 105kDa/110kDa protein 1 | 2.170481618 | 0.017720 | 42/6484 |
| *Hspa1a* | heat shock protein 1A | 3.205741562 | 0.021879 | 45/6484 |
| *Hspa2* | heat shock protein 2 | 2.462584984 | 0.0218791 | 46/6484 |
|  |  |  |  |  |
| **Estrogen signaling pathway** | |  |  |  |
| *Hspa1b* | heat shock protein 1B | 4.094192648 | 1.00E-07 | 5/6484 |
| *Adcy1* | adenylate cyclase 1 | 5.379291925 | 0.000463 | 19/6484 |
| *Hspa1a* | heat shock protein 1A | 3.205741562 | 0.021879 | 45/6484 |
| *Hspa2* | heat shock protein 2 | 2.462584984 | 0.0218791 | 46/6484 |
|  |  |  |  |  |
| **Antigen processing and presentation/ Endocytosis/ Epstein-Barr virus infection/ Toxoplasmosis/ Spliceosome** | |  |  |  |
| *Hspa1b* | heat shock protein 1B | 4.094192648 | 1.00E-07 | 5/6484 |
| *Hspa1a* | heat shock protein 1A | 3.205741562 | 0.021879 | 45/6484 |
| *Hspa2* | heat shock protein 2 | 2.462584984 | 0.0218791 | 46/6484 |
|  |  |  |  |  |
| **Morphine addiction** | |  |  |  |
| *Adcy1* | adenylate cyclase 1 | 5.379291925 | 4.63E-04 | 19/6484 |
| *Adora1* | adenosine A1 receptor | 2.53227807 | 0.00153786 | 22/6484 |
| *Pde4b* | phosphodiesterase 4B, cAMP specific | 2.647289913 | 0.033482 | 48/6484 |
|  |  |  |  |  |
| **cGMP-PKG signaling pathway** | |  |  |  |
| *Atp2b2* | ATPase, Ca++ transporting, plasma membrane 2 | 3.706535376 | 2.29E-06 | 8/6484 |
| *Adcy1* | adenylate cyclase 1 | 5.379291925 | 4.63E-04 | 19/6484 |
| *Adora1* | adenosine A1 receptor | 2.53227807 | 0.00153786 | 22/6484 |
|  |  |  |  |  |
| **Insulin signaling pathway** | |  |  |  |
| *Slc2a4* | solute carrier family 2 (facilitated glucose transporter), member 4 | 4.505247401 | 1.04E-05 | 11/6484 |
| *Gck* | glucokinase | 2.64743733 | 8.49E-04 | 20/6484 |
| *Ppp1r3b* | protein phosphatase 1, regulatory (inhibitor) subunit 3B | 2.911799702 | 5.42E-03 | 31/6484 |
|  |  |  |  |  |
| **Bile secretion** | |  |  |  |
| *Adcy1* | adenylate cyclase 1 | 5.379291925 | 4.63E-04 | 19/6484 |
| *Slc22a7* | solute carrier family 22 (organic anion transporter), member 7 | 2.346475499 | 0.0148102 | 41/6484 |
| *Hmgcr* | 3-hydroxy-3-methylglutaryl-Coenzyme A reductase | 2.405776759 | 0.032158 | 47/6484 |
|  |  |  |  |  |

q values of differentially expressed genes in RNA-sequencing were calculated by Cufflink.

* in all upregulated genes.
